# Precise detection of rearrangement breakpoints in mammalian chromosomes

**DOI:** 10.1186/1471-2105-9-286

**Published:** 2008-06-18

**Authors:** Claire Lemaitre, Eric Tannier, Christian Gautier, Marie-France Sagot

**Affiliations:** 1 Université de Lyon, F-69000, Lyon; Université Lyon 1; CNRS, UMR5558, Laboratoire de Biométrie et Biologie Evolutive, F-69622, Villeurbanne, France; 2 Projet Helix, INRIA Rhône-Alpes, 655 avenue de l'Europe, 38330 Montbonnot Saint-Martin, France

## Abstract

**Background:**

Genomes undergo large structural changes that alter their organisation. The chromosomal regions affected by these rearrangements are called breakpoints, while those which have not been rearranged are called synteny blocks. We developed a method to precisely delimit rearrangement breakpoints on a genome by comparison with the genome of a related species. Contrary to current methods which search for synteny blocks and simply return what remains in the genome as breakpoints, we propose to go further and to investigate the breakpoints themselves in order to refine them.

**Results:**

Given some reliable and non overlapping synteny blocks, the core of the method consists in refining the regions that are not contained in them. By aligning each breakpoint sequence against its specific orthologous sequences in the other species, we can look for weak similarities inside the breakpoint, thus extending the synteny blocks and narrowing the breakpoints. The identification of the narrowed breakpoints relies on a segmentation algorithm and is statistically assessed. Since this method requires as input synteny blocks with some properties which, though they appear natural, are not verified by current methods for detecting such blocks, we further give a formal definition and provide an algorithm to compute them.

The whole method is applied to delimit breakpoints on the human genome when compared to the mouse and dog genomes. Among the 355 human-mouse and 240 human-dog breakpoints, 168 and 146 respectively span less than 50 Kb. We compared the resulting breakpoints with some publicly available ones and show that we achieve a better resolution. Furthermore, we suggest that breakpoints are rarely reduced to a point, and instead consist in often large regions that can be distinguished from the sequences around in terms of segmental duplications, similarity with related species, and transposable elements.

**Conclusion:**

Our method leads to smaller breakpoints than already published ones and allows for a better description of their internal structure. In the majority of cases, our refined regions of breakpoint exhibit specific biological properties (no similarity, presence of segmental duplications and of transposable elements). We hope that this new result may provide some insight into the mechanism and evolutionary properties of chromosomal rearrangements.

## Background

Rearrangements are large scale modifications of the genome, such as inversions or transpositions of DNA segments, translocations between non homologous chromosomes, fusions or fissions of chromosomes, deletions or duplications of small or large portions. Such modifications in the organisation of a genome are not without consequences for the cell and the organism. As a matter of fact, rearrangements have been shown to be responsible for numerous heritable diseases, called genomic disorders. They are further involved in evolution, speciation, and also in cancer (for reviews on all these topics see [1-4]). Although they have been studied for a long time, the underlying mechanisms of such events remain largely unknown, in particular understanding (predicting) their location on the genome. As far as evolutionary rearrangements are concerned, it thus remains an open question to understand what determines their locations. Whereas Nadeau and Taylor suggested in 1984 that rearrangements occur randomly on a genome [5], several observations tend to refute this model and suggest a more deterministic scenario. By comparing genomes of related species, it has thus been suggested that some rearrangements cluster in specific regions, called hotspots [6,7]. A few rearrangement locations have also been found re-used in independent lineages in the course of evolution, indicating again that some regions seem to be more prone to a rearrangement than others [8-10]. In addition, several genomic features, such as segmental duplications or fragile sites, seem to correlate with rearrangement locations [11-13]. However, the nature of the relationship between such features and rearrangements, that is, whether one is a cause or a consequence of the other, remains unknown. To investigate these issues, it is necessary to precisely identify the genomic regions which underwent a rearrangement. The latter is the main motivation of this paper. Of the numerous possible sources of structural variation due to a rearrangement, we deal only with those involving chromosomal regions above a certain size in number of markers (such as genes). The main motivation is that this decreases the risk of false positives, that is, of identifying regions as rearranged while they in fact have been detected as such following a wrong homology assignment. In practice, this means also that we do not deal with duplication or deletion events as those are harder to detect or to properly assign.

One crucial step before analysing the rearrangements and their possible relation with other genomic features is to locate these events on a genome. In the case of two genomes, it is possible to identify conserved regions by comparing the order and orientation of orthologous markers along the genomes. Conserved regions correspond to pairs of segments, one in each genome, that are orthologous and have not been rearranged in either lineage. These are also called *synteny blocks*. Breakpoint regions, or breakpoints for short, are segments that flank the conserved regions. More precisely, a breakpoint is the region between two consecutive synteny blocks on one genome, whose orthologous blocks are rearranged in the other genome (not consecutive or not in the same relative orientations).

A terminological clarification is called for here as the use of the term "breakpoint" to name such rearranged regions can be confusing for two reasons.

The first reason originates from the prefix "break". This suggests a physical break of the DNA (such as a double strand break), and assigns an improper biological meaning to the term. Indeed, the definition of breakpoints is based only on the method developed to identify it. One should therefore be aware that the so-called breakpoint regions have not necessarily been "broken". The region we call breakpoint is located on one genome, and when comparing two genomes, we can usually not decide in which lineage the rearrangement in fact occurred. Suppose for instance that we are comparing the genomes of human and mouse and that the ancestral arrangement of one of the chromosomes is composed of the consecutive synteny blocks *A*, *B *and *C*. Suppose now that the human arrangement is the same as the ancestral one, *ABC*, and that the mouse arrangement is *ACB*. Then, by comparing the human and mouse genomes, the region between *A *and *C *in the mouse genome would appear as a breakpoint, as would the region between *A *and *B *in the human genome. However, neither of these two regions contain the real breakpoint (which is between *A *and *B *in a mouse ancestor), but both are homologous to a broken region.

The second reason why the term "breakpoint" may be confusing originates from its suffix "point". Indeed, most often the location of a breakpoint is not as precise as a point, that is as the position between two nucleotides on a genomic sequence. It concerns rather in general a longer region. This latter is defined as the region between two successive synteny blocks, implying that we have not detected any homology (modelled as a statistically significant enough similarity) for the region to be added to a neighbouring synteny block, or for it to be considered as a new block in itself. However, we do not know *a priori *if this imprecision is due to some biological features created by (or explaining) the break (either the rearrangement itself affects a large region, or many other structural variations occurred before or after the rearrangement in the same region), or to the fact that it is computationally difficult to extend the orthology beyond the extremities of the synteny blocks.

Keeping these considerations in mind, we continue calling such regions breakpoints, for short. We are interested in investigating such breakpoints more in detail. Indeed, in order to analyse the breakpoint sequence and to determine whether breakpoints correlate with some other features of the genome, it is important to precisely locate them. As far as we know, current methods for detecting breakpoints are in fact methods for detecting synteny blocks: they provide the coordinates of the breakpoints only as a byproduct, simply by returning regions that are not found in a conserved synteny. We propose here to go one step further and to extend the synteny blocks by focusing on the breakpoints themselves. It has been previously observed that inside breakpoints, one can often find some smaller blocks of weak similarity that could have been included in the original synteny blocks [14]. We have developed a formal method to precisely locate the breakpoints on a sequenced genome by a comparative approach with related species. Given two genomes, one of which will serve as reference, the core of the method assumes that some synteny blocks have been correctly identified. These delimit regions that are breakpoints but that can be refined in the sense that the blocks could be extended. Thus the regions between which there is no detectable orthology, that is the breakpoints, could be far more precisely and narrowly localised. The method requires that the synteny blocks given as input do not overlap and that their extremities correspond to orthologous sequences between both species. Various methods exist to construct synteny blocks from homologous markers between two sequences, but formal descriptions of these objects are rarer, and no current method can guarantee the simple properties we require. We thus describe our own method to build reliable and formally well described synteny blocks, for which we can guarantee the useful properties.

The method was then applied to mammalian genomic data. The human genome was chosen as reference and compared to two other mammalian genomes: those of the mouse and dog. We end up with a dataset of precise coordinates of mammalian breakpoints on the human genome, which is made publicly available [see Additional files 1 and 2]. By comparison with other published datasets of breakpoint coordinates, we further show that in general, one can extend synteny blocks and refine breakpoints in an efficient enough manner. Finally, we analysed the breakpoint sequences in terms of several genomic features. This identifies some duplications inside the breakpoints and reveals differences with the flanking sequences.

## Methods

### Refining the breakpoints

We start by describing the core of the method, that is the narrowing down as precisely as possible into the breakpoints given a set of synteny blocks.

We are given two sequenced genomes, and the synteny blocks between them. Since we wish to locate all the breakpoints in one genome, the method is not symmetric: one genome is thus the reference and is denoted by *G*_*r*_, while the other genome to which it is compared is denoted by *G*_*o*_. A synteny block is defined by a pair (*A*_*r*_, *A*_*o*_) of coordinates, one (*A*_*r*_) in genome *G*_*r *_and the other (*A*_*o*_) in genome *G*_*o*_, each indicating a chromosome, a start point and an end point. A breakpoint on *G*_*r *_is a region between two synteny blocks that are consecutive on *G*_*r*_, but not on *G*_*o*_. Assuming that the synteny blocks are correct, it is certain that in this region, or in one of its orthologs on the other genome, there has been at least one break due to a rearrangement. The size of the region can vary from several base pairs to several millions of base pairs. As mentioned in introduction, we do not know *a priori *if this imprecision is due to a biological property of the region, or to the fact that orthology has not been detected beyond the extremities of the synteny blocks. We are interested in refining this region as much as possible to eliminate the latter possibility. The refinement of the region is done by aligning the region in-between the two synteny blocks in *G*_*r *_with the regions flanking the orthologs of the blocks in *G*_*o*_. The results of the alignment are then coded into a numerical sequence which is partitioned to identify the narrowed breakpoint.

#### Alignment

**Figure 1:**
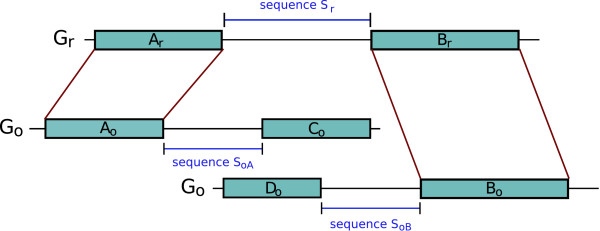
**An example of a breakpoint**. The synteny blocks (*A*_*r*_, *A*_*o*_) and (*B*_*r*_, *B*_*o*_) are consecutive on genome *G*_*r *_but not on *G*_*o*_. It defines a breakpoint on *G*_*r *_between the two blocks. The sequence of the breakpoint is called sequence *S*_*r*_. We define also sequence *S*_*oA *_which flanks block (*A*_*r*_, *A*_*o*_) on *G*_*o *_and is bordered by the next block (*C*_*r*_, *C*_*o*_) on *G*_*o*_. The sequence *S*_*oB *_is defined similarly, flanking block (*B*_*r*_, *B*_*o*_) on *G*_*o *_and bordered by the next block (*D*_*r*_, *D*_*o*_) on *G*_*o*_.

Given two synteny blocks (*A*_*r*_, *A*_*o*_) and (*B*_*r*_, *B*_*o*_) that are consecutive in *G*_*r*_, three sequences of interest are defined (see Figure 1): *S*_*r *_corresponds to the region in *G*_*r *_between *A*_*r *_and *B*_*r*_, *S*_*oA *_and *S*_*oB *_are the sequences flanking *A*_*o *_and *B*_*o *_in *G*_*o *_(respecting the orientation) up to the extremity of the next synteny blocks on *G*_*o*_. Part at least of the sequences *S*_*oA *_and *S*_*oB *_is expected to be orthologous to sequence *S*_*r*_. As an example, in Figure 1, the prefix of sequence *S*_*r *_should be orthologous to the prefix of sequence *S*_*oA *_and its suffix to the suffix of sequence *S*_*oB*_. Depending on the nature of the markers, their extremities may be poorly conserved and the limits of the synteny blocks thus not very clear. The markers at the extremities of the blocks may then be added to the sequences *S*_*r*_, *S*_*oA*_, and *S*_*oB*_. For example, in the case of genes, when their orthology relationship has been assigned based on similarity criteria at the aminoacid level, the orthologous genes may not be alignable on their whole length at the DNA level and thus the extremities of the genes on the genomes may not be orthologous. This can also be due to errors in the prediction of the boundaries of genes. Including the genes at the extremities of the blocks in the sequences thus allows to overcome these problems.

To identify the orthologous relationships between sequence *S*_*r *_and the two sequences *S*_*oA *_and *S*_*oB*_, we perform two local alignments: one of sequence *S*_*r *_against sequence *S*_*oA *_and another of sequence *S*_*r *_against sequence *S*_*oB*_. We use for this the algorithm Blastz [15], after having masked the sequences for repeats with RepeatMasker [16]. The choice of a local aligner like Blastz is motivated by the fact that the sequences are usually (for the major part) intergenic, and therefore in general not well conserved over their whole length, and Blastz has been shown to be more sensitive on such sequences [15]. Large indels, small rearrangements and duplications are possible. The sequences are also often long. These two characteristics call for an algorithm that is both sensitive and fast enough.

Two lists of local alignments, called *hits*, are obtained and mapped onto sequence *S*_*r*_, regardless of their orientations and locations in the sequences *S*_*oA *_and *S*_*oB*_. We expect to have significantly more hits from *S*_*oA *_in sequence *S*_*r *_close to block *A*_*r*_, and more hits from sequence *S*_*oB *_close to *B*_*r*_. In the region in between, in general no clear difference can be made between the amount of hits from *S*_*oA *_and *S*_*oB*_. This defines the *refined breakpoint*.

#### Segmentation

To detect this region in a quantitative manner, the information provided by the hits is coded along the sequence *S*_*r *_giving as result a sequence *I *of discrete values of which three only are possible: -1, 0 and 1. The value is 1 (resp. -1) if position *i *of *S*_*r *_is covered by at least one hit from sequence *S*_*oA *_(resp. *S*_*oB*_) and no hit from sequence *S*_*oB *_(resp. *S*_*oA*_). The value is zero if position *i *is covered by at least one hit from each of the sequences *S*_*oA *_and *S*_*oB*_. The positions covered by no hits are deleted from sequence *I*. Thus sequence *I *has length *n*, the number of positions covered by at least one hit.

The problem we solve is then, given the sequence *I *of -1, 0 and 1's, to find the optimal segmentation of *I *into three segments, such that the first presents an orthology relationship with sequence *S*_*oA*_, the third segment an orthology relationship with sequence *S*_*oB*_, and the segment in between corresponds to the breakpoint. We define two change points *u*_1 _and *u*_2 _over the sequence *I *of length *n*, such that 1 ≤ *u*_1 _≤ *u*_2 _≤ *n*. The sequence is modelled by a piecewise constant function with the values *μ*_1_, *μ*_2 _and *μ*_3 _respectively in the three segments.

We are looking for the two change points *u*_1 _and *u*_2 _that minimise the sum of the squares of the deviations of the data to the model (called the objective function):

(1) $$\begin{array}{ccc} f(u_{1},u_{2})=\sum_{j=1}^{3}\sum_{k=u_{j-1}+1}^{u_{j}}{\left(I_{k}-\mu_{j}\right)}^{2}, & \text{with} & \begin{array}{cc} u_{0}=0 & \begin{array}{cc} \text{and} & u_{3}=n. \end{array} \end{array} \end{array}$$

The values of *μ*_1_, *μ*_2 _and *μ*_3 _are defined as follows:

• $\mu_{1}=\{\begin{array}{l} \begin{array}{ccc} \frac{\sum_{k=1}^{u_{1}}I_{k}}{u_{1}} & \text{if} & \sum_{k=1}^{u_{1}}I_{k}>0 \end{array}\\ \begin{array}{cc} \infty & \text{otherwise} \end{array} \end{array}$

• *μ*_2 _= 0

• $\mu_{3}=\{\begin{array}{l} \begin{array}{ccc} \frac{\sum_{k=u_{2}+1}^{n}I_{k}}{n-u_{2}} & \text{if} & \sum_{k=u_{2}+1}^{n}I_{k}<0 \end{array}\\ \begin{array}{cc} \infty & \text{otherwise} \end{array} \end{array}$

In the middle segment, *μ*_2 _equals zero, thus representing the breakpoint. In the two extreme segments the value of the function is the observed mean of *I *over the segment if the latter has the "correct sign". In order for the first (resp. last) segment to represent an orthology relationship with sequence *S*_*oA *_(resp. *S*_*oB*_), the value of the function must be positive (resp. negative) meaning that it contains more hits with *S*_*oA *_than with *S*_*oB *_(resp. with *S*_*oB *_than with *S*_*oA*_). If the observed mean in the segment has the wrong sign, the value is infinite; it ensures that this segment will not be part of the optimal solution since the contributions over this segment will be infinite.

Observe also that *u*_1 _can be equal to 0, or to *u*_2_, and *u*_2 _can be equal to *n*. This provides the possibility for some segments to be empty, and thus to segment sequence *I *in less than 3 segments.

Since the objective function is additive over the contributions of the positions, a dynamic programming algorithm efficiently provides the optimal partition [17,18]. Notice that, since the number of change points is two, a simple algorithm scanning all possible partitions would be as efficient: the execution time grows with the square of the length *n *of sequence *I*.

#### Speed-up

The problem we solve is, however, more constrained than the classical change-point detection problem. We show that the two change points *u*_1 _and *u*_2 _can be found independently in linear time with the length of sequence *I *instead of using the classical dynamic programming algorithm in *O*(*n*^2^).

**Lemma 1**. *Given the sequence I of size n, such that for all k *∈ {1, *n*}, *I*_*k *_∈ {-1, 0, 1}, *the positions u*_1 _*and u*_2_, *with u*_1 _= *u*_2_, *that minimise the function f *(*u*_1_, *u*_2_) *(see Formula (1)) are such that:*

• $\frac{1}{\sqrt{u_{1}}}\sum_{k=1}^{u_{1}}I_{k}$*is maximal*,

• $\frac{1}{\sqrt{n-u_{2}}}\sum_{k=u_{2}+1}^{n}I_{k}$*is minimal*.

**Proof: **First, by developing the square terms of each sum in function (1), we obtain:

$$f(u_{1},u_{2})=\sum_{k=1}^{n}I_{k}^{2}-\frac{1}{u_{1}}{\left(\sum_{k=1}^{u_{1}}I_{k}\right)}^{2}-\frac{1}{n-u_{2}}{\left(\sum_{k=u_{2}+1}^{n}I_{k}\right)}^{2}$$

The first term is a constant and the other two are independent from each other. Thus *f*(*u*_1_, *u*_2_) is minimal when the two last sums are both maximal, that is when $S_{1}(u_{1})=\frac{1}{\sqrt{u_{1}}}\sum_{k=1}^{u_{1}}I_{k}$ is maximal (since it must be positive), and $S_{2}(u_{2})=\frac{1}{\sqrt{n-u_{2}}}\sum_{k=u_{2}+1}^{n}I_{k}$ is minimal (since it must be negative). However the solution must respect the condition *u*_1 _= *u*_2_. We show next that this condition is always fulfilled.

Let *x*_1 _(resp. *x*_2_) be the position on *I *that maximises *S*_1_(*u*_1_) (resp. minimises *S*_2_(*u*_2_)). Suppose *x*_1 _> *x*_2_.

Then:

$$S_{1}(x_{1})=\frac{1}{\sqrt{x_{1}}}\sum_{k=1}^{x_{1}}I_{k}=\frac{1}{\sqrt{x_{1}}}\sum_{k=1}^{x_{2}}I_{k}+\frac{1}{\sqrt{x_{1}}}\sum_{k=x_{2}+1}^{x_{1}}I_{k}$$

$$S_{2}(x_{2})=\frac{1}{\sqrt{n-x_{2}}}\sum_{k=x_{2}+1}^{n}I_{k}=\frac{1}{\sqrt{n-x_{2}}}\sum_{k=x_{2}+1}^{x_{1}}I_{k}+\frac{1}{\sqrt{n-x_{2}}}\sum_{k=x_{1}+1}^{n}I_{k}$$

• if $\sum_{k=x_{2}+1}^{x_{1}}I_{k}\geq 0$, then $S_{2}(x_{2})\geq \frac{1}{\sqrt{n-x_{2}}}\sum_{k=x_{1}+1}^{n}I_{k}>\frac{1}{\sqrt{n-x_{1}}}\sum_{k=x_{1}+1}^{n}I_{k}=S_{2}(x_{1})$, thus *S*_2_(*x*_2_) is not minimal.

• else $\left(\sum_{k=x_{2}+1}^{x_{1}}I_{k}<0),S_{1}(x_{1})<\frac{1}{\sqrt{x_{1}}}\sum_{k=1}^{x_{2}}I_{k}<\frac{1}{\sqrt{x_{2}}}\sum_{k=1}^{x_{2}}I_{k}=S_{1}(x_{2}\right)$, thus *S*_1_(*x*_1_) is not maximal.

We therefore have that *x*_1 _≤ *x*_2_.     □

#### Statistical test

Whatever the structure of the signal, the method will output the best segmentation of the data into at most three segments. It is therefore important to test if the data are actually structured into three segments, respecting the constraints mentioned above, or if there is no such structure in sequence *I*. In the latter case, the obtained change points do not make statistical sense and we must conclude that we are not able to refine the breakpoint based on the alignments.

The more a given sequence *I *of length *n *is structured into three segments, the lower will be the value of the minimised objective function (that is the sum of the squares of the deviations of the data to the model), and thus the better will be the quality of the fit. What we need to test is therefore whether this fit is significantly better than the one that could be obtained with a non-structured sequence.

The null model is obtained by shuffling sequence *I *and computing for each permutation the value of the objective function corresponding to an optimal segmentation. Since *I *represents the alignment hits, the positions are not independent from one another and the values of 1 and -1 (corresponding to such hits) appear clustered. To take into account this structure in the shuffling operation, we do not shuffle individual positions, but instead blocks of consecutive identical values, given by the extremities of the hits. We accept the null hypothesis that *I *is not structured if more than five percent of the random permutations have a value that is lower than the value obtained by *I*.

### Building the synteny blocks

#### Motivation

We now describe our own method for finding the synteny blocks. The general goal of such a method is to detect unbroken chains of markers which appear in the same order and same orientation in both genomes. Depending on the nature of the markers however, the orthologous relationships can be more or less reliable, and some errors or misleading relationships may disrupt regions of conserved order and orientation. This is why in general more flexible blocks of synteny are constructed.

Methods for identifying orthologous markers and for constructing synteny blocks are numerous in the scientific literature, starting with blocks built from physical or genetic maps of the chromosomes [19], to conserved segments of genes or blocks grouping genomic markers from whole genomic alignments [20-30]. The method to refine breakpoints described in the previous section requires two properties of the synteny blocks. First, they must not overlap on one or the other genome because this would lead to non-existing sequences *S*_*r*_, *S*_*oA *_or *S*_*oB *_since the latter are defined as the sequences that stand between two consecutive blocks. Second, the extremities of the synteny blocks must correspond to an orthology because they are used to define the sequences that will then be aligned. For example, in a block (*A*_*r*_, *A*_*o*_), the sequence at one extremity of *A*_*r *_should be orthologous to the sequence at the corresponding extremity on *A*_*o*_.

**Figure 2:**
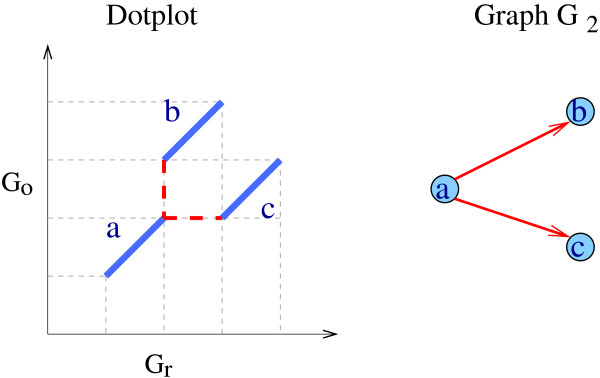
**Example of two conflicting arcs of type I**. On the left side is a dotplot representation of the positions of anchors *a*, *b *and *c *on the genomes *G*_*r *_and *G*_*o*_. They are all on the same chromosome in the two genomes and *d*(*a, b*) = *d*(*a, c*) = 2. On the right-hand side is the corresponding graph G_2 _(*k *= 2). The arcs *ab *and *ac *are conflicting, because the order of the markers of *a*, *b *and *c *in *G*_*r *_is *a, b, c*, whereas it is *a, c, b *in *G*_*o*_.

Few methods for finding blocks available in the literature satisfy these requirements, and those that do so are either very computer intensive or are incompletely described heuristics. For example, GRIMM-synteny [28] builds blocks by clustering markers that are close together, and keeps among the maximal clusters thus detected only those that are bigger than a threshold. The synteny blocks built by GRIMM-synteny may thus overlap. Furthermore, since markers are clustered based on a distance criterion regardless of their order and orientation, the boundaries of a synteny block on any of the two genomes may be defined by two markers which are not orthologous. For instance, in the example of Figure 2, the synteny block composed of the three markers *a*, *b *and *c *would end, according to GRIMM-synteny, with marker *c *in the first genome and marker *b *in the second.

The synteny blocks defined by Sankoff *et al*. in [22] do not overlap, and orthologous markers inside a block appear in the same order in both genomes. However, since the problem they propose to compute synteny blocks is NP-hard, the authors introduce some constraints in order to reduce their original dataset of markers and thus to address the complexity problem. This problem comes from the fact the authors do try to solve the conflicts that may appear and from how they do it. Indeed, when two blocks overlap, the authors attempt to choose which one is the "best" according to some criterion. The one adopted corresponds to maximising the overall number of markers used.

Various other methods based on chaining techniques produce synteny blocks that may overlap [21,24,27,29,31]. They are based on the same principle: two markers are chained if they appear in the same order and orientation in both genomes, and if they stand close enough to one another in terms either of a number of intervening (out of order) markers, or of the physical distance separating them. Only long enough chains are kept, the length corresponding to the number of markers in the chain or the number of nucleotides covered by the chain. None of these methods mentions, and therefore deals with the problem of overlaps and of conflicts between distinct chains.

We then describe our own method for finding the synteny blocks. It is formally well described, and thus it is possible to prove some properties of the blocks found, in particular the ones that are necessary for the refinement of the breakpoints. It takes as input, as all other methods, pairs of homologous markers described by their position and orientation on each genome. Though our approach is closely related to the ones of other published methods (all consist in chaining markers), working with formal definitions of the objects we are looking for guarantees that the synteny blocks we use satisfy the precisely characterised properties we need.

#### Description

We take as input an integer number *k *and a set of anchors between two genomes *G*_*r *_and *G*_*o*_. We call *anchor *a pair of markers, one in *G*_*r *_and one in *G*_*o*_, which are orthologous. We consider only markers that do not overlap in both genomes and that are part of exactly one anchor (no marker is in more than one anchor). If chromosomes are arbitrarily oriented (with a starting point and a reading direction), a marker can be identified by the chromosome it lies in, its position on the chromosome and its orientation (with respect to the starting point of the chromosome). Since we are interested in the relative order of markers on chromosomes, we consider the rank of a marker on a chromosome rather than its physical position on it. An anchor is then identified by a pair of chromosomes, a pair of ranks (the ranks of both markers on each chromosome), and a relative orientation. For instance, let *a *be an anchor, then *a *is identified by (*c*_*r*_, *c*_*o*_, *a*_*r*_, *a*_*o*_, *σ*_*a*_), with *c*_*r *_and *a*_*r *_the, resp., chromosome and rank of the marker on *G*_*r*_, *c*_*o *_and *a*_*o *_the, resp., chromosome and rank of the orthologous marker on *G*_*o*_, and *σ*_*a *_equal to +1 if the two markers have the same orientation, -1 otherwise.

If two distinct anchors *a *and *b *are located on the same chromosome in both species, the *distance *between *a *and *b*, denoted by *d*(*a, b*), is the maximum of the rank differences between *a *and *b *on each genome: if *a*_*r*_, *a*_*o *_(*b*_*r*_, *b*_*o*_) are the ranks of anchor *a *(*b*) on the genomes *G*_*r *_and *G*_*o*_, then *d*(*a*, *b*) = max(|*b*_*r*_- *a*_*r*_|, |*b*_*o*_- *a*_*o*_|).

Thus, if *a *and *b *are consecutive on each genome, the distance between them is one. If two anchors contain markers that are not in the same chromosome in at least one of the species, then the distance is ∞.

Let then G_*k *_be a directed graph, with the anchors as vertices, and an arc between two distinct anchors *a *and *b *with *a*_*r *_<*b*_*r*_, if *d*(*a, b*) ≤ *k*, and either (*a*_*o *_<*b*_*o *_and both anchors have a positive orientation) or (*a*_*o *_> *b*_*o *_and both anchors have a negative orientation). Arcs are identified by the labels of their start and end vertices (anchors). Thus the arc between anchors *a *and *b*, if such exists, is denoted by *ab*.

**Figure 3:**
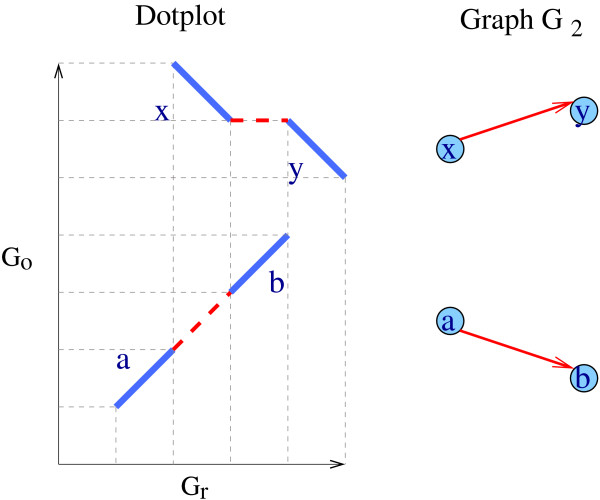
**Example of a conflicting arc of type II**. On the left-hand side is a dotplot representation of the positions of anchors *a*, *b*, *x *and *y *on the *G*_*r *_and *G*_*o*_. Anchors *a *and *b *are on the same chromosome in the two genomes and *d*(*a, b*) = 2, the same for *x *and *y*. On the right-hand side is the corresponding graph G_2 _(*k *= 2). It has two connected components: {*a, b*} and {*x, y*}. The arc *ab *is conflicting because *a*_*r *_<*x*_*r *_<*b*_*r *_and *x *belongs to another component with at least two anchors.

At this step, if *k *is bigger than one, one anchor can be chained to several other anchors, possibly leading to connected components that overlap regarding genomic positions, or to boundaries of connected components that are not defined by a unique anchor. We say in this case that there is a conflict. We define two types of conflict:

• conflict of type I: two arcs *ab *and *cd *belonging to the same connected component of G_*k *_are said to be *conflicting *if the markers of the anchors *a*, *b*, *c*, *d *do not appear in the same order in both genomes (an example where *a *= *c *is given in Figure 2);

• conflict of type II: an arc *ab *in a component *C *is *conflicting *if there exists an anchor *x *whose markers appear between the markers of *a *and *b *in one of the two genomes, and *x *belongs to a connected component of at least *k *vertices, different from *C *(see Figure 3).

Let now H_*k *_be the subgraph of G_*k *_which contains all the non-conflicting arcs of G_*k*_. A *k-block *is a connected component of H_*k *_containing more than *k *vertices. The extremal genomic coordinates of the *k*-blocks define the synteny blocks.

The absence of conflicting arcs of type II ensures that the synteny blocks never intersect. Moreover, the absence of conflicting arcs of type I ensures that in each component, the markers can be totally ordered in both genomes. This yields in particular that one anchor is unambiguously present at each extremity, so we have the required property that the extremities of the blocks in each genome are orthologous.

A polynomial-time computation of the blocks is straightforward from the definition: if *n *is the number of anchors, the computation of the ranks needs the application of a sorting procedure over all anchors in both genomes (in time *O*(*n *log *n*)). Then, the computation of the graph takes time *O*(*n *× *k*), and produces at most *k *× *n *arcs. For each arc, detecting conflicts requires the comparison to at most *k *other arcs or vertices, leading to a total time complexity of *O*(*n *× *k*^2 ^+ *n *log *n*), where *k *is a fixed parameter (we chose *k *= 2 in all our experiments).

#### Discussion on the method

Our method for finding synteny blocks is flexible and outputs totally ordered and non-intersecting *k*-blocks, which is the right entry for the refinement method described in the previous section. Indeed, we build blocks of synteny by chaining orthologous markers that appear in the same order and orientation in the two genomes, but allowing for a number of intervening markers. The maximum degree of flexibility allowed is controlled by one parameter *k*.

This contrasts with most of the other methods which use two parameters [27-29,31], one (denoted by *d*) for the maximum distance allowed between two anchors to be chained and the other (denoted by *S*), for the minimum size of a block to be retained. However, one should fix *d *≤ *S *to prevent a block from lying inside another one. On the other hand, one should fix *d *≥ *S*, to prevent a block of size less than *S*, which we thus consider as irrelevant, from breaking a bigger one. This is why we fix *d *= *S*, and denote it by *k*. Actually, these two parameters *d *and *S *are often assigned the same value when the methods are applied (see for example [6,28,29,31]).

Flexibility is necessary, at least when dealing with orthologous markers whose orthology has been inferred from alignment methods. Indeed, false positives are quite common in this case, particularly in the presence of paralogous sequences. Thus, some false orthology assignments can generate "false" breakpoints, that is regions which have not been rearranged in either of the two lineages. The greater *k *is, the more reliable are the synteny blocks since they are supported by more anchors. However, using a bigger *k *has the drawback of missing small blocks (in number of anchors). The outcome is not only less breakpoints, but also a decrease in the resolution of the remaining breakpoints. In fact, if a block is missed inside a breakpoint, we may not be able to refine it efficiently.

Another outcome of introducing flexibility is that it may produce conflicts. Conflicting arcs represent several chaining possibilities (conflict of type I) or overlapping ones (conflict of type II). Instead of introducing constraints that may not always have an obvious or universal biological meaning, we choose not to solve the conflicts, but instead to discard them. This may seem like a radical solution and, indeed, it produces blocks that are sometimes not as long as they could be if we attempted to solve the conflicts. However, finding the synteny blocks is just one step towards refining the breakpoints and we find preferable to use reliable blocks. The information lost in this initial step will in most cases be recovered in the second step. If removing conflicting arcs implies only the reduction of a block at one of its extremities, the removed extremities on the two genomes will be aligned during the refinement step. On the other hand, if removing conflicting arcs implies missing a whole block, this block will probably not be recovered.

## Results

### Application to two mammalian comparisons

We applied the methods of synteny blocks construction and breakpoint refinement on two pairs of genomes. We detected and refined the breakpoints on the human genome (NCBI35, assembly of May 2004) by comparison, first, with the mouse genome (NCBI m35, assembly of Dec 2005), and then with the dog genome (CanFam 2.0, assembly of May 2005).

For each pairwise comparison, we used the one-to-one orthologous genes available on the Ensembl genome browser [31] as anchors to build the 2-blocks (2-blocks satisfy the definition given in Section 2 for *k-*blocks with *k *= 2) and locate the breakpoints. We applied the refinement method to all the breakpoints, except from those containing a human centromere. We included the sequences of orthologous genes at the extremities of the blocks in the aligned sequences, as suggested in the Method Section. Finally, we applied the permutation test to determine if the change points from the segmentation process are significant. For the human-mouse comparison, starting with 12223 non-overlapping one-to-one human-mouse orthologous gene pairs, we obtained 12018 within a 2-block. We obtained 389 blocks and 366 breakpoints on the human genome, with 355 breakpoints without a human centromere in it. Table 1 gives some statistics on the human-mouse blocks.

**Table 1:** 2-blocks between human and mouse.

length (in bp)	min	max	median	mean
number of orthologous genes inside a 2-block	2	473	13	31
size of the 2-blocks before refinement (in bp)	36,647	79,896,236	2,446,592	6,720,033
size of the breakpoints before refinement (in bp)	1,057	5,311,140	267,891	515,890
size of the breakpoints after refinement (in bp)	21	2,185,434	51,136	128,644

Description of the 2-blocks obtained between human and mouse (before refinement). The 2-blocks satisfy the definition given in Section 2 for *k-*blocks with *k *= 2. Consecutive 2-blocks on both species, with the same relative orientations, have been merged.

**Figure 4:**
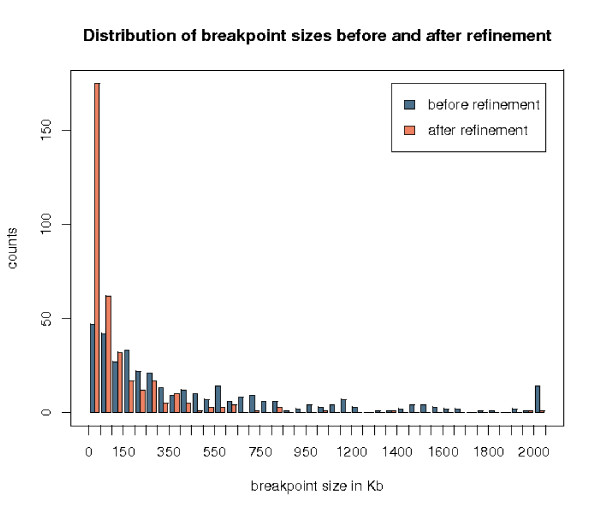
**Size distribution of the 355 breakpoints between human and mouse, before and after refinement**. The last box at 2 000 Kb represents values bigger than 2 Mb.

Out of the 355 refined breakpoints, only one is not significant for the permutation test of the segmentation. After further investigation, it appeared that this breakpoint corresponds to a mouse duplication, with the entire sequence *S*_*r *_aligned with both sequences *S*_*oA *_and *S*_*oB*_. Figure 4 shows a histogram with the sizes of the breakpoints before and after refinement. On average, a breakpoint is reduced by 552 Kilobases and we obtained after refinement 171 (48%) breakpoints less than 50 Kb in size [see Additional file 1].

Concerning now the human-dog comparison, starting from 12839 non-overlapping one-to-one pairs of orthologous genes, we obtained 12663 within a 2-block. This led to 272 blocks and 249 breakpoints, with 240 without a human centromer in it. Table 2 gives some statitics on the blocks.

**Table 2:** 2-blocks between human and dog.

length (in bp)	min	max	median	mean
number of orthologous genes inside a 2-block	2	443	20	46.5
size of the 2-blocks before refinement (in bp)	8,382	154,604,141	3,212,701	9,945,063
size of the breakpoints before refinement (in bp)	5,963	3,108,685	241,505	437,558
size of the breakpoints after refinement (in bp)	66	1,224,273	33,140	86,074

Description of the 2-blocks obtained between human and dog (before refinement).

The permutation test of the segmentation was significant for all the 240 refined breakpoints. On average, a breakpoint is reduced by 506 Kilobases, and we obtained after refinement 145 (60%) breakpoints less than 50 Kb in size [see Additional file 2].

### Comparison with alignment-based methods

We compared the breakpoint sizes obtained by our method with those of other publicly available datasets of breakpoints. We used three datasets of breakpoints between human and mouse, all of them based on whole genome alignment methods. The first two are obtained with the algorithm GRIMM-synteny, the first one is a pairwise comparison of human and mouse [28] while the second is a multiple comparison between human, mouse and rat [32]. We call them, respectively, GRIMM2 and GRIMM3. The third one is retrieved from the Ensembl genome browser, version 34 and we call it ENSEMBL. The method used in this case is succinctly described on the Ensembl web page [33]: it consists, starting with Blastz whole genome alignments retrieved from the UCSC genome browser [34], in chaining alignments that are distant by no more than a certain *max_gap *in number of bp, and in discarding chained blocks which span less than *min_len *in size. It is similar to GRIMM-synteny, and to our synteny block generation method, except that conflicts are not taken into account. The breakpoints we have defined with our own method are referred to as the REFINED breakpoints, or REFINED for short.

For the three datasets, we computed the breakpoints as the regions between two consecutive synteny blocks on the human genome that are not consecutive on the mouse genome. We eliminated breakpoints containing a human centromere, and when synteny blocks overlap on the human genome, we considered the intersection as the breakpoint.

We started by globally comparing the distribution of the breakpoint sizes between the different datasets. The REFINED breakpoints are globally smaller than the breakpoints from the other datasets, with an average length of 129 Kb versus 364, 454 and 1513 Kb for, respectively, GRIMM2, GRIMM3 and ENSEMBL (Table 3). We compared each dataset with ours, using the Wilcoxon rank sum test. The differences are highly significant with the respective p-values of 2. 085*e *– 14, < 2. 2*e *– 16 and 4. 977*e *– 05, when compared with GRIMM2, GRIMM3 and ENSEMBL.

**Table 3:** Comparison of the distributions of breakpoint sizes between the four datasets.

length	min	max	median	mean
REFINED	21	2,185,434	51,136	128,644
GRIMM2	313	5,418,383	155,816	364,199
GRIMM3	2,490	4,953,520	267,609	454,490
ENSEMBL	2	82,331,123	106,534	1,513,770

The numbers are expressed in base pairs.

**Figure 5:**
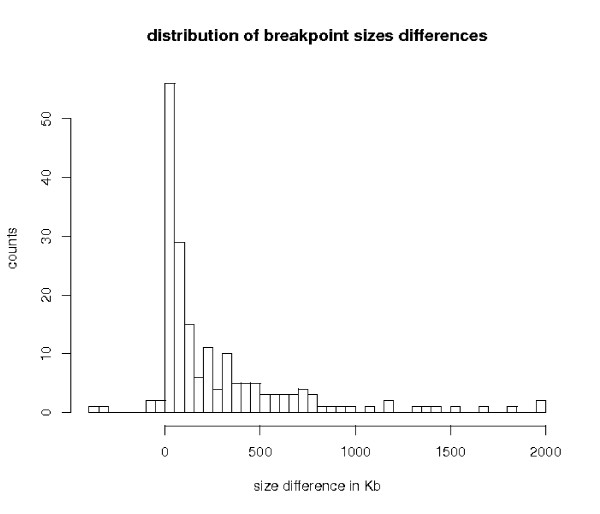
**Distribution of the differences in breakpoint sizes pairwisely computed between GRIMM3 and our dataset (186 common breakpoints)**. A positive value means that the breakpoint size is bigger in GRIMM3 than in our dataset. The last box at 2 000 Kb represents values bigger than 2 Mb.

Since all datasets do not contain the same number of breakpoints (the REFINED breakpoints set contains 354 breakpoints, whereas GRIMM2, GRIMM3 and the ENSEMBL datasets contain 246, 306 and 200 respectively), one could argue that we do not compare the same breakpoints, and that the length difference observed is only due to dataset-specific breakpoints. In order to test this hypothesis, we compared the length of the breakpoints which are common to both the REFINED and the GRIMM3 sets. The coordinates of the GRIMM3 breakpoints lie on a different assembly version of the human genome (NCBI33 assembly). Using the Ensembl identifier of the orthologous genes bordering our breakpoints (as landmarks), we could unambigously identify 186 common breakpoints. For each breakpoint, we calculated the length difference between GRIMM3 and the REFINED breakpoints. The distribution of the length differences is plotted in Figure 5. On average, the GRIMM3 breakpoints are 276 Kb bigger than the REFINED ones, which is significant to a paired Wilcoxon test (pvalue of < 2. 2*e *– 16).

Observe that the REFINED breakpoints are computed on a more complete human genome assembly, and some gaps in the former assemblies could have prevented the detection of some synteny blocks and led to their absence from the GRIMM3 dataset.

**Figure 6:**
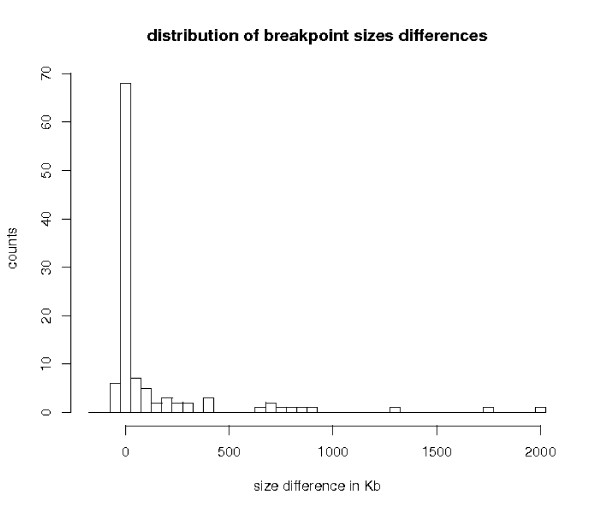
**Distribution of the differences in breakpoint sizes pairwisely computed between ENSEMBL and our dataset (108 common breakpoints)**. A positive value means that the breakpoint size is larger in the ENSEMBL than in our dataset. The last box at 2 000 Kb represents values bigger than 2 Mb.

We made a similar pairwise comparison with the ENSEMBL dataset, for which the breakpoints lie on the same assembly version as ours. We thus eliminated this potential assembly effect. We identified 108 common breakpoints and for each one, we calculated the length difference between the ENSEMBL and the REFINED breakpoints. The average length difference remains positive, meaning that the REFINED breakpoints are smaller than the ENSEMBL ones (mean difference of 143 Kb). The differences are less marked, as one third of the compared breakpoints differ by less than 1 Kb (see the distribution of the length differences in Figure 6).

We should however be careful with the latter results, as the ENSEMBL dataset appeared not reliable in general. First, it contains less breakpoints than the other datasets (only 200), although it was obtained with less stringent parameters than GRIMM2 (for example, the minimum size of a synteny block is 100 Kb for ENSEMBL and 1 Mb for GRIMM2) which should give more breakpoints. Moreover, the distribution of their length is unusual with some breakpoints very small and some very large. In particular, the biggest breakpoints (bigger than 20 Mb) make us believe that some synteny blocks may have been missed inside. Finally, if we compute the genomic coverage of the synteny blocks, we obtain that only 76.8% (2.317 Gb) of the genome is covered by the ENSEMBL blocks, whereas the coverage is much larger for the other datasets (89.6%, 89.5% and 86.7% for GRIMM2, GRIMM3 and the 2-blocks before refinement respectively).

### Genomic features in the breakpoints

**Figure 7:**
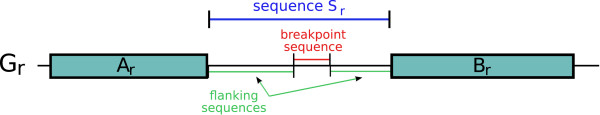
**Schematic representation of a breakpoint and its flanking sequences**. The original breakpoint (before refinement) lies between synteny blocks *A*_*r *_and *B*_*r *_on genome *G*_*r*_, its sequence is called *S*_*r*_. The *breakpoint sequence *after refinement, is represented in red. The *flanking sequences *(showed in green) are defined as the sequences of sequence *Sr *that are not part of the breakpoint region. We consider in this analysis breakpoints whose sequence (in red) spans more than 10 Kb, and for which at least one flanking sequence spans more than 10 Kb.

Interestingly, even after refinement, the majority of the breakpoints are still big regions and are not reduced to a point. We wanted to test whether these regions have particular characteristics with respect to those inside the sequences newly appended to the synteny blocks by the refinement method. We thus compared the sequences of the breakpoints bigger than 10 Kb detected in the human-mouse comparison with their flanking sequences, defined as the regions outside the refined breakpoints which are not in the original synteny blocks (see Figure 7). We measured the coverage of each sequence in whole genome local alignments, human segmental duplications and transposable elements.

**Figure 8:**
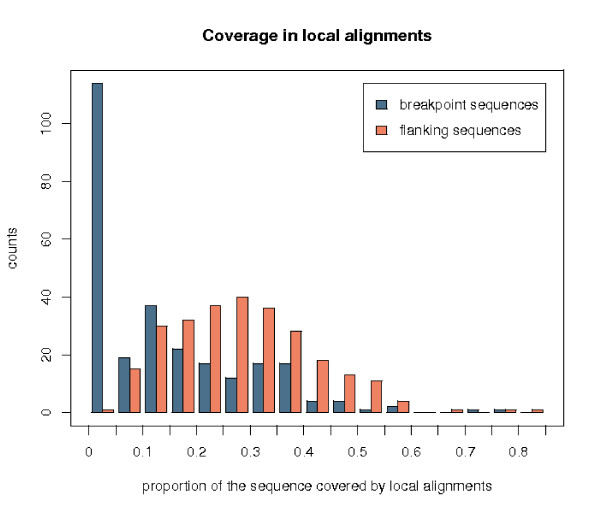
**Comparison of the distributions of sequence coverage by local alignments**. Breakpoint sequences are represented in blue, whereas the flanking sequences are in orange.

The motivation for measuring the presence (and amount) of whole genome (human-mouse) local alignments in a human sequence is that this is indicative of the similarity between the latter sequence and any part of the mouse genome. The whole genome local alignments were taken from the chain-net alignments files available on the UCSC genome browser [34]. The method is described in [26]. It appeared that breakpoints are depleted in local alignments (average coverage of 14%) with respect to their flanking sequences (28%) and the overall coverage of the genome (36%) (see the average distributions in Figure 8). This is statistically significant using a paired Wilcoxon test (pvalue of 2. 2*e *– 16). Notably, 42% of the breakpoint sequences (114 breakpoints) have less than 5% of their length covered by a local alignment. Observe that these alignments are obtained from whole genome comparisons and are not necessarily part of a synteny block. It suggests that a number of breakpoints, spanning sometimes several hundreds of kilobases, do not show any similarity with any part of the other genome. Either they are very fast evolving sequences, or they correspond to insertions of new sequences in the human genome or to deletions in the mouse one. While some breakpoints show no similarity with any part of the genome, others show similarity with several parts, either in the human or in the mouse genomes, sometimes preventing us from being able to refine them. This is the case, for instance, of segmental duplications.

Segmental duplications, also called Low Copy Repeats, are large duplications (typically greater than 1 Kb), with a high percentage of identity (typically more than 90%), that are found in a small number of copies (as opposed to transposable elements) and often clustered in mammalian genomes. Recently [10-12], it has been shown that breakpoints and duplications tend to co-localise. Two different hypotheses have been suggested to explain this trend: either the duplications caused the rearrangements by similarity-dependent mechanisms, such as non allelic homologous recombination (NAHR); or the duplications accumulated at the same places because of an inherent fragility of these genomic regions. We observed here a similar trend. Breakpoints are overall more covered by segmental duplications (average coverage of 27%) than their flanking sequences (average coverage of 15%). This is again statistically significant using a paired Wilcoxon test (pvalue of 2. 22*e *– 9). Notably, 34 breakpoint sequences are almost entirely covered by segmental duplications (coverage greater than 90%).

Other duplicated sequences, that do not have necessarily the same properties as segmental duplications (in terms of length or similarity level) have been found associated with rearrangements. The duplicated sequences were found at each extremity of an inverted segment, in the opposite orientation [35-37]. An alternative mechanism to NAHR was suggested, which is that the duplication may have appeared as a consequence of the rearrangement, as a fill-in of the gaps resulting from staggered break ends. Thanks to our refinement step, we can suggest candidates for this situation: this is the case when the human breakpoint sequence coincides with the mouse duplicates located in the two corresponding breakpoints on the mouse genome, namely in sequences *S*_*oA *_and *S*_*oB *_(Figure 1). Indeed, when a breakpoint aligns with both sequence *S*_*oA *_and sequence *S*_*oB*_, it corresponds to the middle segment of the numerical sequence *I *which contains many zeros. We can thus easily detect these special cases. For the human-mouse comparison, we obtain 41 breakpoints which present such characteristic (more than half of the middle segments of the numerical sequence *I *correspond to a zero, meaning these positions are covered by hits from both sequence *S*_*oA *_and *S*_*oB*_). In 36 cases, the breakpoints are absent from the human-dog comparison, suggesting that the involved rearrangements occurred in the mouse lineage, and further arguing for a relation between duplication and rearrangement events.

Finally, we observe a similar trend for transposable elements as for whole genome local alignments and segmental duplications. Overall, breakpoints are richer in transposable elements than their flanking sequences (average coverage of 53% against 48% respectively), paired Student test significant (pvalue of 6. 15*e *– 8). When we distinguish for the different types of transposable elements (SINEs, LINEs, LTR elements and DNA transposons), no significant difference is observed, except for LTR elements (average coverage of 11.4% in breakpoints versus 8.6% in the flanking sequences, pvalue of 2. 21*e *– 5 for a paired Wilcoxon test).

## Discussion and conclusion

With the availability of whole genome sequences, one would have hoped to be able to compare genomes at the nucleotide level and thus to locate evolutionary events, such as rearrangements, up to a base pair. Such precision is required, for instance, in order to identify potential footprints left on the sequence by a rearrangement. However, current available breakpoint data lack such precision. The goal of the method we presented in this paper is to obtain breakpoints as precise as possible. Our strategy to achieve this is divided in two steps: the first one identifies reliable blocks and the second refines the breakpoint regions in between using the information (corresponding to the orthologous sequences to align) obtained in the previous step.

Having reliable synteny blocks is a requirement to the refinement method, and is the reason why we chose to deal with markers that are genes. Since they are functional elements, they are usually more conserved (and so more reliable) than intergenic DNA. Moreover, orthology assignments for genes are computed at the aminoacid level, which is even more conserved. The genomic coverage of orthologous genes is less extensive than the coverage of the alignments obtained by a whole genome comparison method which may detect similarity even in the non coding parts of the genome. It thus leads to synteny blocks which present also a low genomic coverage. However, the synteny blocks can be extended beyond the genes, and eventually we may reach a better coverage than with whole genome alignments. Nevertheless, we wish to emphasise that the method described here is not restricted to this kind of synteny blocks, and that it can also be applied on synteny blocks originally obtained from whole genome alignments for instance.

The advantage of proceeding in two steps is that by reducing the search space for homology in the first step, we can look for weaker similarity inside the breakpoint regions in the second step. This is one of the reasons why our method gives more precise breakpoints than whole genome alignment methods. Indeed, the latter operate in a single step, and require the use of stringent enough parameters to avoid obtaining blocks which are not orthologous. However, one drawback is then that they miss weak similarity inside the breakpoints.

We have shown here that this similarity does exist inside breakpoints and synteny blocks may indeed be refined, as was already pointed out by [14], and we propose a quantitative method to this end.

The second argument accounting for the gain in precision of our method is based on the number of genomes compared. With the availability of an increasing number of fully sequenced genomes, methods using more than two genomes to identify synteny blocks are often privileged. However, we chose to develop a pairwise method. The motivation is to gain even further precision. Comparison of the breakpoint sizes show that 3-way blocks (such as obtained by GRIMM3) give bigger breakpoints than those obtained with a pairwise comparison (such as in GRIMM2) using the same method, even when adopting less stringent parameters. This comes from the fact that the GRIMM3 anchors are three-way, meaning that one anchor represents an orthologous marker in each of the three species. This leads to more confident anchors than pairwise ones, but it also reduces the set of anchors and thus the size of the synteny blocks.

**Figure 9:**
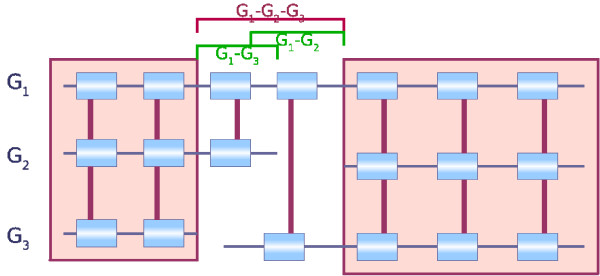
Example of two closely located breakpoints that overlap position-wise.

**Figure 10:**
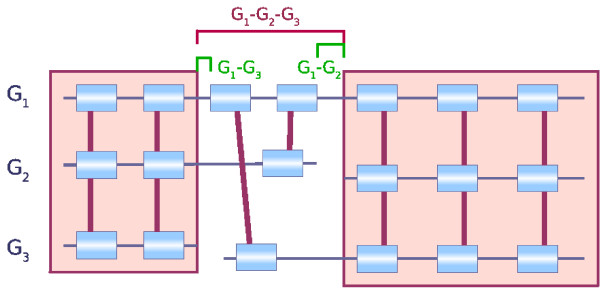
**Example of two closely located breakpoints that do not overlap position-wise**. The two breakpoints appear fused when using a multiple comparison and which pairwise comparisons enable to clearly identify as two distinct breakpoints.

Although multiple comparisons could be useful to compute the synteny blocks, we argue that to refine breakpoints, a pairwise method is preferable. Indeed, this enables to be more sensitive in the detection of homology. Moreover, it also allows to discriminate between one or several rearrangement events in a seemingly common region. For example, suppose two breakpoints are very close to each other on the human genome, one being observed by comparison with the mouse genome, and the other with the dog genome. Investigating the two breakpoints by pairwise (independent) comparisons allows to determine whether they overlap position-wise as in Figure 9. Using a multiple comparison, if the two breakpoints are too close to each other (the distance between them is less than the minimum size of a synteny block), only one breakpoint may be identified. An example of this is given in Figure 10 where two distinct breakpoints are perceived as fused when doing a multiple comparison while pairwise comparisons enable to separately identify the two. It thus seems preferable to, first, identify precisely breakpoints between two genomes, and then to compare them with the breakpoints obtained in other species comparisons while trying to infer their evolutionary relationship. This strategy could be useful to estimate the amount of rearrangement re-use in independent lineages. As an example, we obtained five cases where a mouse breakpoint and a dog breakpoint do not overlap and are less than 50 Kb apart on the human genome (10 cases when the threshold is set to 100 Kb, 39 cases for 300 Kb).

Finally, comparison of the breakpoints with their flanking sequences confirms previous studies of rearrangement breakpoints where loss of similarity, enrichment in segmental duplications and in transposable elements were revealed [9-12,14,26]. Moreover, it shows that breakpoints are actually regions which can be distinguished from the remaining of the genome, and reinforces the belief that breakpoints are indeed regions, and not single points.

## Authors' contributions

CL developped and implemented the method. All authors participated in discussions and writting of the paper.

## Supplementary Material

Additional file 1 **Dataset of the breakpoints between human and mouse**. The file contains the coordinates of the breakpoints obtained with the method described in the paper, with the human genome as reference, compared with the mouse genome. The breakpoints lie on the human genome (assembly version NCBI35). The file format is plain text, each line corresponds to one breakpoint, and there are three columns (chromosome, beginning and end positions of the breakpoint) separated by a space. Click here for file

Additional file 2 **Dataset of the breakpoints between human and dog**. The file contains the coordinates of the breakpoints obtained with the method described in the paper, with the human genome as reference, compared with the dog genome. The breakpoints lie on the human genome (assembly version NCBI35). The file format is plain text, each line corresponds to one breakpoint, and there are three columns (chromosome, beginning and end positions of the breakpoint) separated by a space. Click here for file
